# Editorial: Autism Spectrum Disorders (ASD)-Searching for the Biological Basis for Behavioral Symptoms and New Therapeutic Targets

**DOI:** 10.3389/fnins.2016.00607

**Published:** 2017-01-09

**Authors:** Benjamin Gesundheit, Joshua P. Rosenzweig

**Affiliations:** Cell-El Ltd.Jerusalem, Israel

**Keywords:** Autism Spectrum Disorders (ASD), HLA antigens, behavior, intelligence, sensory thresholds, genetic syndromes, autoimmune diseases

The frequency of Autism Spectrum Disorders (ASDs) is increasing with a 30% reported increase in pediatric prevalence from 2012 to 2014 in the U.S. until present rates of about 1 in 68 children or 1.5% of children in the U.S. (Corcoran et al., 2015). Yet, little is known about the etiology of this spectrum. As of now, ASD is diagnosed based on a series of behavioral tests. The challenge for researchers is to try to uncover the biological basis for these typical behaviors in order to improve diagnosis and identify potential targets for treatment. A multidisciplinary approach to understanding the biological basis for the behavioral symptoms is necessary in order to move forward. This includes analysis of the current animal models for ASD and their suitability, reviewing behavioral, immunological, immunogenetic, and epigenetic research, reassessing clinical diagnostic tools, and surveying radiological, pathological, and serological records for clues.

With over 500 animal models available with varying construct validity and face validity, and a variety of behavioral tests for animals (Kazdoba et al., 2016; three chamber, T maze, elevated plus maze) and for humans (ADOS, ADIR, CARS, ABC) and still no FDA approved effective treatments for the core symptoms of autism, much more needs to be done to understand the behavioral features of autism and their underlying etiology. Thillay et al. used EEG to record 12 adults diagnosed with ASD and age matched controls performing a visual target detection task. Their data suggests that patients diagnosed with autism overreact to stimuli coming from an unclear context, which matches their sense of being overwhelmed by incoming data, and that they are unable to control cortical activity according to varying levels of uncertainty. Parallel to the response to an uncertain context, Corbett et al. investigated the difference in stress response to interaction with peers as well as the role of sensory sensitivity. They found that children with ASD showed significantly higher cortisol levels than their typically developing control group. These results indicated that increased cortisol was associated with increased sensory sensitivity and enhanced stress. Schauder and Bennetto integrated the empirical literature on sensory processing in ASD with those papers that investigate neural response to sensory stimuli. Sensory symptoms start demonstrating relationships with adaptive functioning and language proficiency in the early years. Therefore, in order to generate a multidisciplinary approach to sensory processing in ASD, it is critical to integrate the sensory symptoms and neuroscience perspectives. Internal and external stimuli can elicit two different categories of responses, an excitatory response, and an inhibitory response. Frye et al. reviewed biological abnormalities shared by ASD and epilepsy and found that autism and epilepsy are associated with comparable aberrations that may alter the excitatory to inhibitory balance of the cortex. They suggested that these parallels may explain the high prevalence of epilepsy in ASD and the elevated prevalence of ASD features in individuals with epilepsy. Instead of looking at other disorders that are similar to autism such as epilepsy, Zachor and Ben-Itzchak set out to investigate whether specific medical conditions in ASD are associated with unique behavior profiles. They found two unique medical behavioral subtypes in ASD that affect inherited traits of cognition and/or autism severity. Crespi analyzed the innovative hypothesis that autism is actually a disorder of high intelligence. They propose that looking at both intelligence and autism studies together could provide unique insights into the neurological and genetic causes of high mental abilities.

Efforts at twin studies, identifying HLA associations, specific genes, single nucleotide variants (SNVs) or single nucleotide polymorphisms (SNPs), and hotspots for copy number variations (CNVs) in autism have yielded limited but promising results so far. In light of the fact that Rett Syndrome, Fragile X, and other genetic syndromes comorbid with ASD have been shown to be associated with epigenetic modifications the theory that epigenetic mechanisms might potentially be associated with the etiology of ASD deserves more attention. Due to mounting evidence indicating immune involvement in the etiology of autism, Torres et al. looked at common genetic variants found in HLA and KIR immune genes, particularly HLA genes on chromosome 6 and KIR genes on chromosome 9. They show that for HLA class I alleles, frequencies are significantly increased by more than 5% over control populations. They also found that three activating KIR genes have increased frequencies of 15, 22, and 14% in the autism populations, and that there is a 6% increase in total activating KIR genes in autism when compared to controls. Similarly, Gamliel et al. performed a study comparing the KIR:HLA frequencies in ASD children with those of their healthy parents. They found a higher frequency of HLA-C2 allotypes among the fathers, while its corresponding ligand 2DS1, was higher in the maternal group. Francis et al. also looked at genetic variants, yet they focused on the receptor genes of oxytocin and vasopressin, since studies have reported significant associations between these genes and ASD diagnosis and ASD-related phenotypes. They found associations between vasopressin receptor single nucleotide polymorphisms (SNPs) and specific oxytocin receptor SNPs and diagnosis and behavioral profile. Instead of looking at specific genetic variants, Ansel et al.) analyzed gene expression studies from the past decade and came up with a comprehensive list of genes that were found to be dysregulated in ASD children as compared to typically developing controls.

Ornoy et al. emphasized that an ASD diagnosis is often an important clinical presentation of some well-known genetic syndromes in men. They reviewed these syndromes and also looked at the role of the most important prenatal factors affecting the fetus throughout pregnancy, which may be associated with ASD as well as maternal autoimmune diseases, and infections, which are associated with ASD. Similarly, Nardone and Elliott reviewed the growing evidence for a complex interaction between immune system activation in the mother during pregnancy and epigenetic control in the brain of the fetus that may help generate an autistic phenotype. They looked at this particularly because of molecular studies that have highlighted the role of epigenetics in brain development as a process susceptible to environmental influences and potentially causative of ASD. Looking at both the immune state of the mother and the fetus, Grether et al. found that in both maternal and newborn there was a significantly lower risk of ASD associated with higher levels of Toxo IgG. These results support previous studies indicating that immune factors during early development may be relevant to the etiology of ASD. Many serological studies aimed at identifying any abnormalities in the blood of children with ASD have yielded conflicting results (Kalra et al., 2015). Nevertheless, various inflammatory cytokines and immunological markers reflecting immune dysfunction have been documented in ASD. Preliminary studies even suggest a correlation between certain antibodies and clinical severity (Ashwood et al., 2011). In order to counteract the heterogeneity of ASD, larger studies with broader screening of immune factors are necessary.

Though there is still much work to do in uncovering the biological basis for ASD, patterns are beginning to emerge. The combination of serological data with genetic data enables researchers to isolate pathways that demonstrate particular association with ASD. The precise mechanisms between these networks and the behavioral symptoms have yet to be fully elucidated. However, larger studies with more unified diagnostic inclusion criteria and multidisciplinary testing will hopefully yield further hints toward identifying the underlying mechanism of ASD.

## Author contributions

BG contributed substantially to the conception of the work and revised it critically. JR contributed substantially to the conception, design, and analysis of the work. JR also drafted the work.

### Conflict of interest statement

The authors declare that the research was conducted in the absence of any commercial or financial relationships that could be construed as a potential conflict of interest.
